# Biophysical Stimuli as the Fourth Pillar of Bone Tissue Engineering

**DOI:** 10.3389/fcell.2021.790050

**Published:** 2021-11-09

**Authors:** Zhuowen Hao, Zhenhua Xu, Xuan Wang, Yi Wang, Hanke Li, Tianhong Chen, Yingkun Hu, Renxin Chen, Kegang Huang, Chao Chen, Jingfeng Li

**Affiliations:** ^1^ Department of Orthopedics, Zhongnan Hospital of Wuhan University, Wuhan, China; ^2^ Wuhan Institute of Proactive Health Management Science, Wuhan, China; ^3^ Department of Orthopedics, Union Hospital, Tongji Medical College, Huazhong University of Science and Technology, Wuhan, China; ^4^ Department of Orthopedics, Hefeng Central Hospital, Enshi, China

**Keywords:** biophysical stimuli, mesenchymal stem cells, osteoinductive mechanisms, biophysical transduction, osteogenesis

## Abstract

The repair of critical bone defects remains challenging worldwide. Three canonical pillars (biomaterial scaffolds, bioactive molecules, and stem cells) of bone tissue engineering have been widely used for bone regeneration in separate or combined strategies, but the delivery of bioactive molecules has several obvious drawbacks. Biophysical stimuli have great potential to become the fourth pillar of bone tissue engineering, which can be categorized into three groups depending on their physical properties: internal structural stimuli, external mechanical stimuli, and electromagnetic stimuli. In this review, distinctive biophysical stimuli coupled with their osteoinductive windows or parameters are initially presented to induce the osteogenesis of mesenchymal stem cells (MSCs). Then, osteoinductive mechanisms of biophysical transduction (a combination of mechanotransduction and electrocoupling) are reviewed to direct the osteogenic differentiation of MSCs. These mechanisms include biophysical sensing, transmission, and regulation. Furthermore, distinctive application strategies of biophysical stimuli are presented for bone tissue engineering, including predesigned biomaterials, tissue-engineered bone grafts, and postoperative biophysical stimuli loading strategies. Finally, ongoing challenges and future perspectives are discussed.

## 1 Introduction

After trauma, bone tissue shows self-healing property, but this ability is limited for critical bone defects (average diameter over 2 cm in humans) caused by serious injury, tumor excision, or other orthopedic diseases (Lopes et al., 2018). Bone healing failure, which occurs in 5–10% of all patients with bone fracture, generally causes delayed union (healing process over 3 months) or non-union (healing process over 9 months without obvious bone regeneration in the first 3 months) (Zura et al., 2016; Wojda and Donahue, 2018). Autologous bone grafting is currently the gold standard for the healing of critical bone defects because it provides three critical components: an osteoconductive substrate, osteoinducive signals, and preosteoblastic cells (Yong et al., 2020). However, the strategy fails to meet clinical requirements because of limited autografts, potential donor site complications (such as infections, chronic pain, and bleeding), and the risk of graft failure (Roseti et al., 2017; Yang et al., 2018).

Bone tissue engineering has been becoming an ideal strategy to replace autologous bone grafting, and it is composed of three pillars to emulate the basic components of autografts: biomaterial scaffolds, bioactive molecules, and stem cells (Huang et al., 2020). Bioactive molecules are generally in the form of recombinant growth factors or small molecular bioactive peptides to provide osteoinductive properties (Yang et al., 2018). But the delivery of bioactive molecules shows some limitations: 1) initial burst release, 2) declined biological activity, 3) high therapeutic dosage, and 4) potential side effects (Krishnan et al., 2017; Bertrand et al., 2020). Therefore, another pillar showing osteoinductive properties needs to be incorporated into bone tissue engineering for bone regeneration or bone healing.

Biophysical stimuli have attracted great attention for bone regeneration because of their great promise as the fourth pillar of bone tissue engineering. From Wolff’s law to Frost’s “mechanostat theory”, a plethora of evidence verifies that bone is a mechanosensitive tissue (Tyrovola 2015; Haffner-Luntzer et al., 2016; Qin E. C. et al., 2020). Multiple bone cells that respond to biophysical stimuli include osteocytes, osteoblasts, osteoclasts, bone lining cells, and mesenchymal stem cells (MSCs) (Steward and Kelly, 2015; Stewart et al., 2020). In bone tissue engineering, the osteogenic differentiation of MSCs is the most important process. Therefore, this review focuses on the osteoinductive effects of biophysical stimuli toward MSCs. Biophysical stimuli with osteoinductive properties can be categorized into three groups depending on their physical properties: internal structural stimuli, external mechanical stimuli, and electromagnetic stimuli.

**FIGURE 1:**
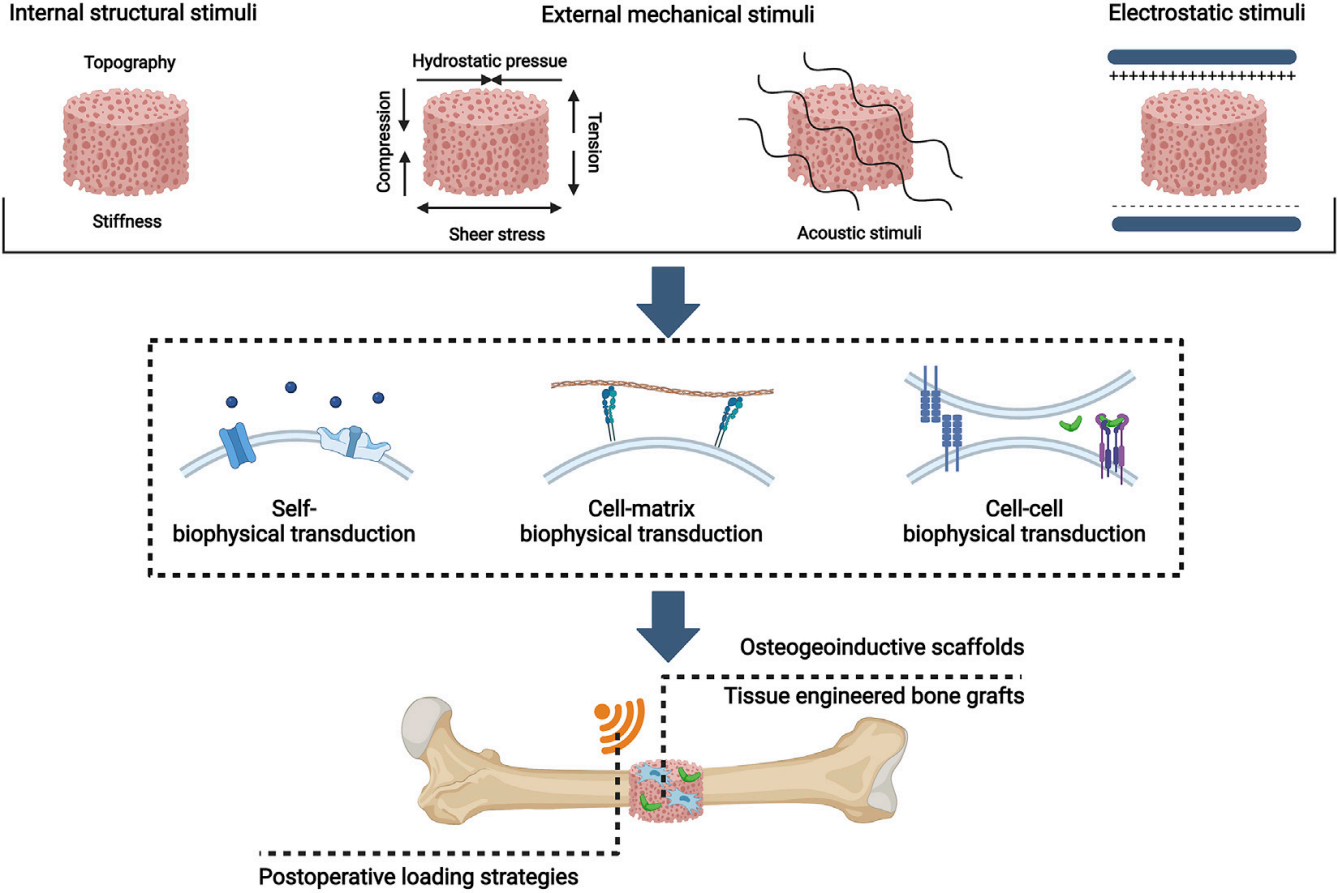
Overview of biophysical stimuli for bone tissue engineering: distinctive patterns, osteoinductive mechanisms, and bone tissue engineering applications. Created with BioRender.com.

External mechanical stimuli were proposed as the fourth pillar of bone regeneration by Lopes et al. (2018). Here we suggest that the concept can cover even more comprehensive forms of biophysical stimuli. In this review, we first update the fourth pillar of bone tissue engineering as biophysical stimuli and summarize distinctive biophysical stimuli with their osteoinductive windows for MSC osteogenesis, including internal structural stimuli, external mechanical stimuli, and electromagnetic stimuli. Then, a novel concept of biophysical transduction (a process of sensing, transmission, and regulation) that incorporates mechanotransduction and electrocoupling is proposed to interpret the osteoinductive mechanisms of biophysical stimuli for the osteogenic differentiation of MSCs. And biophysical stimuli, depending on sensing mechanisms, can be divided into self-biophysical transduction, cell-matrix transduction, and cell-cell biophysical transduction. Moreover, the application strategies of biophysical stimuli as the fourth pillar of bone tissue engineering are presented, which include preconstructed scaffolds with osteoinductive properties, tissue engineered bone grafts (TEBGs), and postoperative biophysical stimuli loading strategies. (Figure 1). This review aims to propose a novel and comprehensive concept that biophysical stimuli show potential to be used as the fourth pillar of bone tissue engineering.

## 2 Distinctive Biophysical Stimuli for Bone Tissue Engineering

**FIGURE 2:**
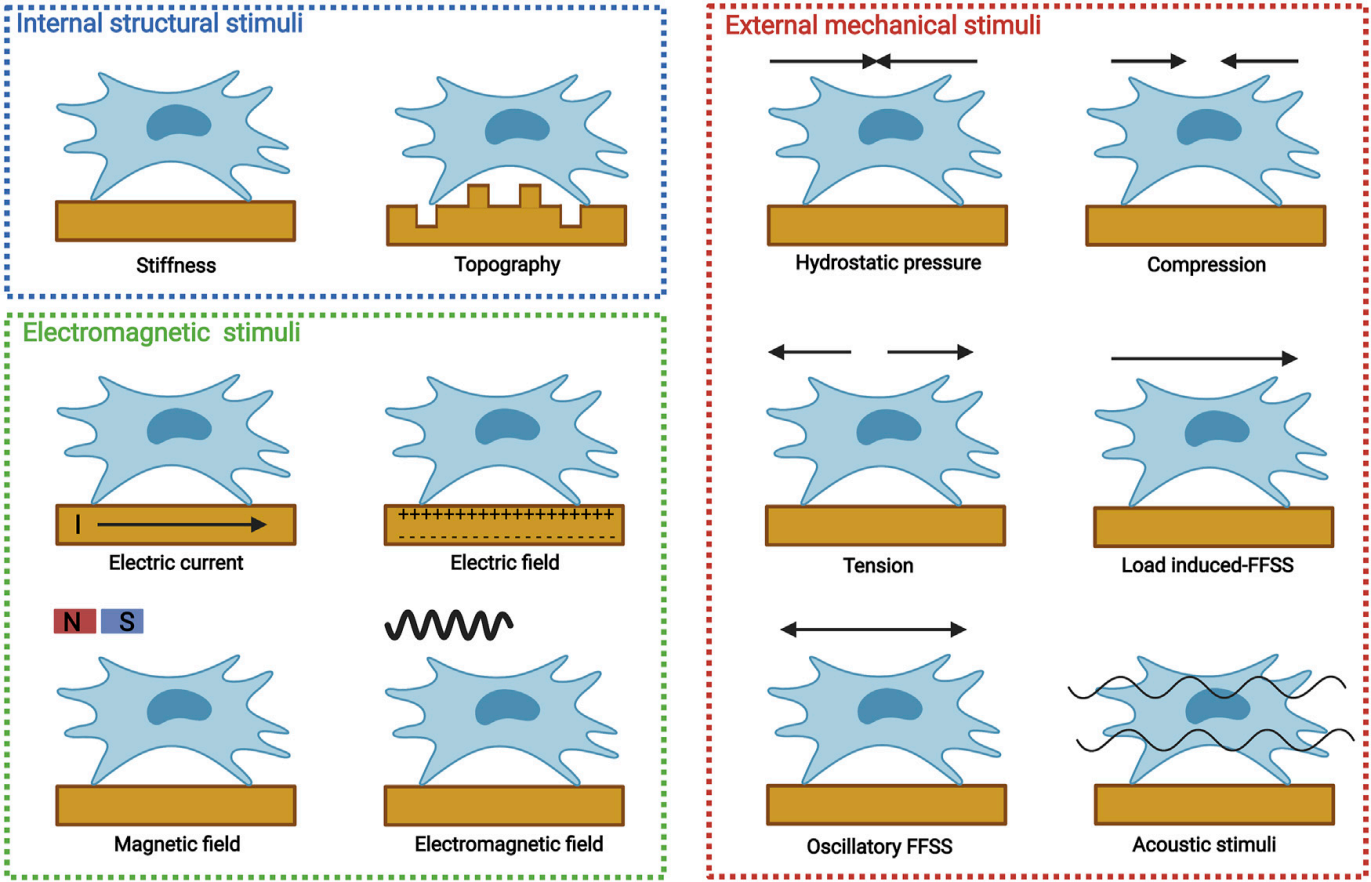
Distinctive biophysical stimuli for bone tissue engineering, including internal structural stimuli (stiffness and topography), external mechanical stimuli [hydrostatic pressure, compression, tension, load induced-fluid flow shear stress (FFSS), oscillatory FFSS without load, and acoustic stimuli], and electromagnetic stimuli (electric current, electric field, magnetic field, and electromagnetic field). Created with BioRender.com.

MSCs can be obtained from various tissues, including bone marrow-derived MSCs (BMSCs), periosteum-derived stem cells (PDSCs), adipose-derived stem cells (ADSCs), and periodontal ligament stem cells (PDLCs). For bone tissue engineering, MSCs are introduced to biomaterials either by direct encapsulation or indirect recruitment. Biophysical stimuli could modulate various MSC processes, including migration, proliferation, and differentiation. Distinctive biophysical stimuli with different parameters may result in different MSC specifications. Biophysical stimuli for osteogenic differentiation should be limited by one or several parameters, which can be termed as osteoinductive windows. Depending on physical properties, biophysical stimuli can be categorized into internal structural stimuli, eternally mechanical stimuli, and electromagnetic stimuli (Figure 2).

### 2.1 Internal Structural Stimuli

Internal structural stimuli are derived from matrix microenvironment where MSCs survive and grow. In human, distinctive tissues show different mechanical properties, among which matrix stiffness and topography determine the fate of MSCs.

#### 2.1.1 Matrix Stiffness

Matrix stiffness is the rigidity or elasticity of the three dimensional (3D) microenvironment. MSCs show different cell shapes when loaded on the surface of collagens with distinctive stiffness (Engler et al., 2006). In specific microenvironment with different matrix stiffness, they transfer from the initially round shape to a branched (0.1–1 kPa), spindle (8–17 kPa), or polygonal (25–40 kPa) shape, which then determines their commitment for neurogenic, myogenic, or osteogenic differentiation (Engler et al., 2006). The results can be also supported by the fact that spread, flattened, and adherent MSCs undergo osteogenic differentiation, whereas unspread and round MSCs undergo adipogenic differentiation (McBeath et al., 2004). However, in a 3D microenvironment, the influence of cell shape transfers to nanoscale integrin binding and the rearrangement of cell adhesion ligands, which could stimulate the osteogenic differentiation of MSCs by contractility (Huebsch et al., 2010). In addition, 3D matrix with a stiffness of 11–30 kPa predominantly stimulates osteogenesis (Huebsch et al., 2010).

#### 2.1.2 Topography

Topography is another mechanical property related to cell adhesion. According to the patterning size, topography exerts effects on different levels, including macroscale colony level (>100 µm), microscale cell level (0.1–100 µm), and nanoscale receptor level (1.0–100 nm), among which nanotopography influences the commitment of MSCs (Prè et al., 2013). Various nanopatterns (such as nanopits, nanorods, nanopillars, or nanocolumns) can be modified on the surface of biomaterials for the osteogenic differentiation of MSCs, and specific osteoinductive parameters (shape, diameter, spacing, height, or depth) vary greatly among these nanopatterns and different fabrication techniques (Dobbenga et al., 2016). However, all osteoinductive windows of different nanotopographies show nanoscale-controlled disorder, a nanopattern that is not completely random and not highly ordered (Prè et al., 2013). Dalby et al. first fabricated five nanotopographies on the surface of polymethylmethacrylate embossed with nanopits (diameter 120 nm, depth 100 nm): highly ordered hexagonal array (center–center spacing 300 nm), highly ordered square array (center–center spacing 300 nm), disordered square array with a controlled displacement of 20 nm (center–center spacing 300 ± 20 nm), and disordered square array with a controlled displacement of 50 nm (center–center spacing 300 ± 50 nm) (Dalby et al., 2007). All highly ordered groups and completely random groups fail to sufficiently induce the osteogenic differentiation of MSCs, but both disordered nanotopographies with controlled displacement show osteoinductive properties; those with a controlled displacement of 50 nm are superior to those with a controlled displacement of 20 nm in terms of osteocalcin (OCN), osteopontin (OPN), and bone nodule contents (Dalby et al., 2007). Zhang et al. utilized nanorods to explore the osteoinductive properties of nanotopography with controlled disorder (Zhou J. et al., 2016). They fabricated five nanopatterns with different interrod spacings (302.7 ± 10.5, 137.2 ± 7.5, 95.9 ± 3.8, 66.8 ± 4.1, and 32.6 ± 2.7 nm). And they found that the group with interrod spacings over 137 nm impedes MSC osteogenesis, the group with interrod spacings below 96 nm facilitates osteogenic differentiation, and the 66.8 ± 4.1 nm group shows preferable osteoinduction (Zhou X. et al., 2016). The above results suggested that the interspacing of nanopattern determines the osteoinductive windows of nanotopography. And the modification of nonopits may need relatively large interspacing because the spacing area supports cell adhesion, whereas the modification of nanorods or nanopillars needs relatively small interspacing because they support cell adhesion. Although nanopillars with different heights (Sjöström et al., 2009; McNamara et al., 2011; Sjöström et al., 2013) or nanopits with distinctive diameters (Lavenus et al., 2011) show different osteoinductive properties, nanopattern interspacing changes with height. Thus, whether or not the height or diameter of nanopatterns truly controls the osteogenic differentiation of MSCs remains unknown, and further studies should make spacing constant while changing other parameters.

### 2.2 External Mechanical Stimuli

External mechanical stimuli are derived from external forces, which could exert effects on MSCs continuously or cyclically. External mechanical stimuli that show osteoinductive properties include hydrostatic pressure (HP), compression, tension, load-induced fluid flow shear stress (FFSS), oscillatory FFSS, and acoustic stimuli.

#### 2.2.1 Hydrostatic Pressure

MSCs reside in a fluid-filled microenvironment. Thus, HP affects the fate of MSCs, which may exert homogenous compression to MSCs. Huang et al. used cyclic HP (0.5 MPa, 0.5 Hz) by a perfusion bioreactor and found that the sinusoidal profile could promote osteogenic differentiation, but the proliferation is spoiled (Huang and Ogawa, 2012). HP with high magnitude does not accord with physiological HP; hence, high-magnitude HP may be limited for regenerative medicine. From a physiological perspective, MSCs in the bone marrow are exposed to static intramedullary pressure (approximately 4 kPa), which increases to 50 kPa when exposed to external mechanical stimuli, and stem cells in the perivascular space and Haversian channels may experience 300 kPa pressure. Thus, researchers further compared the osteogenic effects of three HPs (10, 100, and 300 kPa) with different frequencies (0.5, 1, and 2 Hz) and durations (1, 2, and 4 h) and found that HP with 300 kPa and 2 Hz produces the most effective osteoinductive property (Stavenschi et al., 2018). However, the collagen synthesis and mineral deposition are similar among different groups, showing that HP with 10 kPa is sufficient to induce MSC osteogenesis (Stavenschi et al., 2018). Reinwald et al. found that intermittent HP (270 kPa, 1 Hz, 60 min/day, 21 days) promotes the osteogenic differentiation of MSCs when loaded onto poly (E-caprolactone) (PCL) scaffolds (Reinwald and El Haj, 2018). Altogether, cyclic or intermittent HP with magnitude 10–300 kPa induces MSC osteogenesis.

#### 2.2.2 Compression

In addition to HP, compression (physiological strain from 0.2 to 0.4%) is induced on the vertical direction of the force when natural bone is compressed (Al Nazer et al., 2012). Depending on loading pattern, compression can be classified into uniaxial compression and equiaxial compression, both of which could induce the osteogenic differentiation of MSCs when they are limited by specific parameters. The stiffness of biomaterials is enhanced to promote osteogenesis when scaffolds are exposed to compression (Baumgartner et al., 2018). Among parameters describing compression, the magnitude of compression strain determines the fate of compressed MSCs. However, osteoinductive compression strain magnitude varies greatly because of different compression devices, durations, and biomaterials. For relatively high compression strain (≥1.5%), whether or not high-magnitude compression stimulates osteogenesis remains controversial (Haudenschild et al., 2009; Sittichokechaiwut et al., 2010; Aziz et al., 2019; Schreivogel et al., 2019). It was revealed that 5% compression could induce the osteogenic differentiation of MSCs loaded onto polyurethane scaffolds without biochemical cues, which show comparable osteoinduction with dexamethasone (Sittichokechaiwut et al., 2010). However, Horner et al. adopted four compression strains (5, 10, 15, and 20%) and found that osteogenic markers decrease and chondrogenic markers increase in a magnitude-dependent manner (Horner et al., 2018). Haudenschild et al. also found that ±5% bulk strain with 5% offset stimulates chondrogenic differentiation (Haudenschild et al., 2009). Indeed, high compression strain inhibits the expression of Runt-related transcription factor 2 (RUNX2), but the expression of bone Morphogenetic Protein-2 (BMP-2) is interestingly upregulated (Schreivogel et al., 2019). Thus, one potential explanation for this discrepancy is that abundant culture medium blocks the effects of mechanosensitive autocrine factors, which impede osteogenic differentiation. Schreivogel et al. further explored the effects of autocrine factors by improving the number of scaffolds containing MSCs and reducing the volume of culture medium; results showed that 5 and 10% compression could promote the osteogenic differentiation of MSCs (Schreivogel et al., 2019). Therefore, high-magnitude compression may induce osteogenesis by mechanosensitive autocrine factors, such as BMP-2, and the ratio of cell number and medium volume may determine the fate of MSCs. Compared with high-magnitude compression, low-magnitude compression could directly promote the expression of osteogenic markers. A previous study seeded MSCs to monetite calcium phosphate scaffolds and then subjected them to compression (0.4%, 0.1 Hz) (Gharibi et al., 2013). After 2 h stimulation, some immediate-early response genes are activated, which promote the expression of other genets (such as RUNX-2) to induce the proliferation and osteogenic differentiation of MSCs (Gharibi et al., 2013). Ravichandran et al. adopted low compression strains (0.22, 0.88, and 1.1%) and found that the 0.22% group induces more alkaline phosphatase (ALP) and calcium than the other groups (Ravichandran et al., 2017). These studies indicate that although compression with high magnitude stimulates osteogenesis by mechanosensitive autocrine factors, the precise ratio of cell number to medium volume is difficult to control. Thus, physiological compression (0.2–0.4%) shows great promise for MSC osteogenesis.

#### 2.2.3 Tension

When natural bone is pulled, tension is also induced on the vertical direction of the force. Depending on the loading pattern, tension could be divided into uniaxial compression and equiaxial compression. Different from compression, tension with high strain (such as 5%) promotes osteogenic differentiation and inhibits adipogenic differentiation (Li et al., 2015a). Tensile strain-inducing cell culture plates are generally used to study the effects of tension. In these two dimensional (2D) models, MSCs are initially seeded on the cell culture plates coated with the matrix, and then the plates are subjected to tension strain, which indirectly exerts tension to MSCs. Thus, the matrix should show great ability for cell adhesion, and type I collagen has been widely used for luxuriant arginine–glycine–aspartate (RGD) peptide (Sumanasinghe et al., 2009). Lohberger et al. seeded MSCs on type I collagen-coated cell culture plates, which were then subjected to continuous tension (10%, 0.5 Hz), and the tension-loaded groups show improved expression of osteogenic genes, higher calcium deposition, and more ALP when compared with the unstimulated groups (Lohberger et al., 2014). (Zhao et al., 2010) compared the effects of 0.3 Hz tension with different strains (9, 12, and 15%) and found that the 12% tension group induces robust osteogenic response (Zhao et al., 2021). To explore the effects of tension in a 3D microenvironment, a novel uniaxial tension bioreactor was designed to exert tensile forces (10%, 0.5 Hz for 7 days with 4 h each day) to fibrin hydrogels seeded with MSCs (Carroll et al., 2017). Results show that tension could promote the intramembranous ossification and impede the adipogenic differentiation of MSCs (Carroll et al., 2017). Therefore, the osteoinductive window of tension is mainly determined by tension strain, and 5–15% magnitude could stimulate MSC osteogenesis.

#### 2.2.4 Fluid Flow Shear Stress (Perfusion and Rotation)

FFSS is another external mechanical stimulus generated by the load of compression or tension. Load-induced FFSS could change cell shape. One classic 2D model to explore the effect of load-induced FFSS is parallel-plate flow chamber (Yourek et al., 2010; Dash et al., 2020). Using this model, it was confirmed that short-term continuous FFSS (9 dynes/cm^2^) for 24 h could promote the osteogenic differentiation of MSCs without chemically osteoinductive molecules (Yourek et al., 2010). However, for long-term intermittent FFSS, low-magnitude FFSS (such as 10 mPa, namely, 0.1 dynes/cm^2^) is sufficient to induce osteogenesis (Dash et al., 2020). However, these strategies fail to emulate the natural ECM microenvironment; thus, various bioreactors, including rotation and perfusion bioreactors, have been developed to generate FFSS. Perfusion bioreactors have been widely used to explore the osteoinductive effects of FFSS (Filipowska et al., 2016). In the absence of biochemical cues (such as dexamethasone), FFSS (1 ml/min) provided by perfusion bioreactors for 16 days could dramatically promote the mineralization of MSCs within decellularized matrix/Ti meshes (Datta et al., 2006). Bjerre et al. (2008) used a perfusion bioreactor to dynamically culture MSCs seeded in silicate-substituted tricalcium phosphate scaffolds and found that FFSS (0.1 ml/min) for 21 days could promote the proliferation and osteogenic differentiation of MSCs. Filipowska et al. established an intermittent model (2.5 ml/min for three times with 2 h per section) by using perfusion bioreactors to stimulate MSCs seeded in gelatin-coated polyurethane scaffolds and found that intermittent protocols can induce MSC osteogenesis (Filipowska et al., 2016). During dynamic perfusion culture, the expression of type X collagen is upregulated, suggesting that endochondral and intramembranous ossification participates in osteogenesis (Moser et al., 2018). Therefore, the osteogenic window of load-induced FFSS is mainly determined by interchangeable pressure or flow rate, and load-induced FFSS with pressure > 0.2 dynes/cm^2^ shows osteoinductive properties (Yong et al., 2020).

#### 2.2.5 Oscillatory Fluid Flow Shear Stress (Microvibration and Nanovibration)

Oscillatory FFSS is another FFSS generated by oscillatory displacement without strain, which is the microscale or nanoscale form of vibration (Stewart et al., 2020; Birks and Uzer, 2021). According to amplitude, vibration can be classified into microvibration (≤50 µm) and nanovibration (<100 nm).

Microvibration is vibration with amplitude ≤ 50 µm, magnitude < 1 g, and frequency 1–100 Hz (Wu et al., 2020). Frequency may determine the osteoinductive window of microvibration. Cashion et al. revealed that microvibration with a low frequency (1 Hz) induces chondrogenesis, whereas relatively high frequency (100 Hz) promotes osteogenesis (Cashion et al., 2014). Thus, low-magnitude high-frequency vibration (LMHFV) (magnitude < 1 g, frequency 20–90 Hz) is generally used for osteogenesis (Steppe et al., 2020). 50 Hz LMHFVs with different magnitudes (0.1, 0.3, 0.6, and 0.9 g) were used to stimulate PDLCs, and it was found that all groups promote osteogenic differentiation, but LMHFV with 0.3 g peaks the osteoinduction (Zhang et al., 2015). Some studies revealed that LMHFV stimulates MSC osteogenesis in a frequency-dependent response. One study revealed that horizontal vibration at 100 Hz causes higher osteoinduction than that at 30 Hz (Pongkitwitoon et al., 2016). A previous study observed that 800 Hz microvibration promotes higher biomineralization and osteogenic marker expression than 0, 30, and 400 Hz, but long-term stimulation of microvibration (30 min/day, 14 days) with frequencies of 30 and 400 Hz inhibits osteogenesis (Chen et al., 2015). Thus, stimulation duration and loading time also influence the osteogenic differentiation of MSCs. One study revealed that microvibration (60 Hz, 1 h/d for 5 days) inhibits the mineralization and osteogenic differentiation of MSCs (Lau et al., 2011), whereas another study showed that microvibration (30 Hz, 45 min/day for 21 or 40 days) could promote MSC osteogenesis (Prè et al., 2013). These results suggest that the osteoinductive window of microvibration can be determined by frequency, duration, and single loading time. For 30 Hz vibration, long-term duration may be effective to osteogenic differentiation. For vibration with frequencies over 60 Hz, single loading time should be limited with 30 min, and short-term duration (<7 days) may be superior for osteogenesis.

Nanovibration refers to vibration with nanoscale amplitude (<100 nm). It was confirmed that nanovibration (frequency 1,000 Hz, amplitude 10–14 nm) could promote the osteogenic differentiation of MSCs in 2D condition (Nikukar et al., 2013). Another study showed that nanovibration (frequency 1,000 Hz, amplitude 10–14 nm) could stimulate the osteogenic differentiation of MSCs seeded in 3D collagen hydrogels (Tsimbouri et al., 2017). Thus, the osteoinductive window of nanovibration is a frequency of approximately 1,000 Hz and amplitude of 10–20 nm.

#### 2.2.6 Acoustic Stimuli

Acoustic stimuli, according to frequency, can be categorized into infrasound (<20 Hz), audible sound (20–20,000 Hz), and ultrasound (>20,000 Hz). Therapeutic acoustic stimuli are generally termed as ultrasound with frequency varying from 0.7 to 3.3 MHz and low or high intensity (Zhang et al., 2017). When loaded to tissue, ultrasound could convert energy to heat via thermal effect, which may cause irreversible damage. Some nonthermal effects induced by ultrasound, including cavitation, acoustic microstreaming, acoustic radiation force, the spread of surface waves, and oscillatory FFSS may modulate the commitment of MSCs. (Esfandiari et al., 2014; Padilla et al., 2014).

Low-intensity pulsed ultrasound (LIPUS) (intensity 30–100 mW/cm^2^, frequency 1.5 MHz, and duty cycle 20% or 100%) is a preferential strategy to reduce the thermal effect for regenerative medicine. This strategy has been approved by the United States Food and Drug Administration for the treatment of fresh fractures and established non-union (de Lucas et al., 2020). Depending on duty cycle, continuous LIPUS (100%) can also be used. However, one research revealed that LIPUS with 20% duty cycle shows higher osteoinductive properties toward ADSCs than LIPUS with 50% duty cycle (Yue et al., 2013). Thus, LIPUS may be more effective than cLIPUS. Using PDLCs, the osteoinduction of LIPUS was explored, and it was found that LIPUS could stimulate osteogenic differentiation and upregulate osteocalcin, Runx2, and integrin 1, and that LIPUS with an intensity of 90 mW/cm^2^ is more effective for osteoinduction than LIPUS with an intensity of 30 or 60 mW/cm^2^ (Hu et al., 2014). Zhou et al. further improved the intensity of LIPUS and found that LIPUS with an intensity of 150 mW/cm^2^ is more effective than LIPUS with intensities of 20, 50, 75, and 300 mW/cm^2^ (Zhou X. et al., 2016). These results suggest that the osteoinductive window of LIPUS is primarily determined by duty cycle and intensity, and 20% duty cycle and 90–150 mW/cm^2^ intensity may be optimal for the osteogenic differentiation of MSCs.

Pulsed focused ultrasound (intensity 133 W/cm^2^, frequency 1 MHz, and duty cycle 5%) is another acoustic stimulus model with relatively minimizing thermal effect that is characterized by short term and high intensity (de Lucas et al., 2020). It could induce MSC homing (Burks et al., 2018), but whether or not it induces the osteogenic differentiation of MSCs remains unclear.

### 2.3 Electromagnetic Stimuli

Electromagnetic stimuli are also important biophysical cues that could exert effects on the fate of MSCs. Depending on physical properties, electromagnetic stimuli can be further classified into magnetic stimuli in the form of magnetic field and electric stimuli in the form of electric field and electric current.

#### 2.3.1 Electric Current

Natural bone physiologically generates electric stimuli on account of non-centrosymmetric collagen after mechanical stress, which supports bone development and repair (Khare et al., 2020). Thus, external electric stimuli can be applied to MSCs for regenerative medicine. Alternating electric current is an effective external electric stimulus that induces the osteogenic differentiation of MSCs (Creecy et al., 2013). In one study, MSCs were seeded on an indium-tin-oxide-coated glass and then subjected to alternating electric current (5–40 µA, 5–10 Hz, 1–24 h/day) for 21 days without exogenous biochemical osteogenic molecules, and results showed that all setups of alternating electric current could stimulate osteogenic differentiation and inhibit chondrogenic and adipogenic differentiation (Wechsler et al., 2016). In addition, alternating electric current (10 µA, 10 Hz, 6 h/day) could reach optimized osteogenic effects (Wechsler et al., 2016). Direct current is another form of electric current, but its osteogenic effects without electric field remain unknown, which needs further research.

#### 2.3.2 Electric Field

Electric field is another effective external electric stimulus to promote MSCs toward osteogenic differentiation. According to the generation pattern, electric field can be categorized into direct current electric field, capacitively coupled electric field, and inductively coupled electric field (Thrivikraman et al., 2018). When MSCs are exposed to electrical stimuli, the membrane potential could be altered, and hyperpolarization stimulates osteogenesis (Murillo et al., 2017; Bhavsar et al., 2019). In addition, the configuration of plasma receptors could be modulated for osteogenic differentiation (Murillo et al., 2017). Furthermore, cytoskeletal elongation and nuclear orientation could be changed by electric field for osteogenesis (Khaw et al., 2021).

Among various parameters, electric field intensity may determine the osteoinductive windows. Using osteogenic differentiation medium, a previous study applied an electric field (2 mV/mm, 60 kHz, 40 min/day) to induce MSCs and found that the electric field could induce a delayed osteogenic differentiation of MSCs (Esfandiari et al., 2014). However, Hronik-Tupaj et al. reported that a 2 mV/mm, 60 kHz electric field promotes chondrogenesis (Hronik-Tupaj et al., 2011). The discrepancy may be interpreted as the utilization of osteogenic molecules, which could synthetically direct electric field to promote osteogenesis. One research revealed that electric field (0.36 mV/mm, 10 Hz) alone fails to induce osteogenic differentiation but dramatically promotes MSC osteogenesis when osteogenic sulfated hyaluronan derivative is added (Hess et al., 2012). Therefore, electric field with low intensity may not promote MSCs toward osteoblasts but could synthetically improve the osteoinductive properties of biochemical molecules.

Different from low-intensity electric field, high-intensity electric field could directly promote the osteogenic differentiation of MSCs. For instance, when MSCs are subjected to electric field (100 mV/mm, 1 h/day), osteogenic differentiation occurs, and osteoinductive effects could be maintained even when the electric field is removed (Hess et al., 2012; Eischen-Loges et al., 2018). Khaw et al. utilized two electric fields (100 and 200 mV/mm) to MSCs without biochemical osteogenic supplements, and found that both electric fields could promote the osteogenic differentiation of MSCs, and the 200 mV/mm electric field was optimized (Khaw et al., 2021). Ravikumar et al. designed an electric field device where a static potential (15 V) was loaded to parallel electrodes with a space of 15 mm (Ravikumar et al., 2017). MSCs were seeded to HA-CaTiO_3_ composites and then exposed to electric field for 10 min/day without osteogenic molecules, and results showed that the electric field could dramatically improve the osteogenic markers (Ravikumar et al., 2017). These studies suggest that an electric field with an intensity over 100 mV/mm may be needed for MSC osteogenesis without biochemical osteogenic molecules.

#### 2.3.3 Magnetic Field

Exposure of MSCs to magnetic field may directly deform their plasma membrane, which causes cytoskeleton remodeling, improves cell viability, and promotes differentiation (Santos et al., 2015). Magnetic biomaterials should be introduced to culture systems, including magnetic particles and substrates, to enhance the effects of magnetic field. Boda et al. fabricated a series of hydroxyapatite-Fe_3_O_4_ magnetic substrates with different magnetization and then applied a periodic magnetic field (100 mT) to these magnetic substrates seeded with MSCs, and they found that all magnetic substrates combined with magnetic field could promote the osteogenic differentiation of MSCs (Boda et al., 2015). Magnetic particles can be also used to transform magnetic stimuli into mechanical stimuli. Magnetic particles coupled with magnetic field (14.7 or 21.6 mT) were used to stimulate ADSCs, and it was found that low-density magnetic field (14.7 mT) with intermittent short-term exposure (2 days) favors adipogenic differentiation, whereas high-density magnetic field (21.6 mT) promotes osteogenesis in all exposure profiles including continuous or intermittent long-term exposure (7 days) and intermittent short-term exposure (2 days) (Labusca et al., 2020). To further control the effects of magnetic particles, magnetic particles are generally functionalized by antibodies or peptides, which could directly exert pico-newton level forces to mechanosensitive plasma membrane receptors under magnetic field, which could then induce the osteogenic differentiation of MSCs (Kanczler et al., 2010; Hu et al., 2013). For example, Henstock et al. used either the antibody of transmembrane ion channel stretch-activated potassium channel (TREK-1) or RGD peptide to functionalize magnetic nanoparticles (Henstock et al., 2014). Then, an oscillating magnetic field (25 mT) was loaded to collagen hydrogels containing MSCs and functionalized magnetic nanoparticles to generate a force of 4 pN, and results showed that functionalized magnetic nanoparticles could promote matrix mineralization (Henstock et al., 2014). The osteogenic window of magnetic field may be determined by magnetic flux density (21.6–100 mT), and the quantity and functionalization of magnetic nanoparticles affect their osteogenic effectiveness.

#### 2.3.4 Electromagnetic Field

Electromagnetic field is a combination of electric and magnetic fields, and pulsed electromagnetic field (PEMF) has been clinically used to delay osteoporosis and bone fracture repair (Sun et al., 2009). When loaded to MSCs, PEMF without osteogenic molecules inherently induces osteogenic differentiation and impedes angiogenic differentiation, and the osteogenic effects can be further enhanced by additional osteogenic molecules (Ongaro et al., 2014; Lu et al., 2015). For example, Arjmand et al. seeded ADSCs to PCL nanofibrous scaffolds, which were then exposed to PEMF (1 mT, 50 Hz) with or without osteogenic medium (Arjmand et al., 2018). Results revealed that the osteogenic effects of PCL scaffolds with PEMF are comparable with those of PCL scaffolds with osteogenic medium and that PCL scaffolds with PEMF and osteogenic medium show enhanced osteogenic differentiation (Arjmand et al., 2018). PEMF has been also verified to stimulate MSC proliferation (Tsai et al., 2009; Sun et al., 2010). Therefore, PEMF shows great promise for bone tissue engineering.

Among various parameters describing PEMF, magnetic flux density and frequency may determine the osteoinductive window of PEMF. For magnetic flux density, Jazayeri et al. compared two PEMFs (0.1 and 0.2 mT) and found that the expression of osteogenic markers, such as RUNX2 and OCN, is higher under 0.2 mT PEGF than under 0.1 mT PEGF (Jazayeri et al., 2017). Esposito et al. used a device that could generate 1.8–3 mT PEGF to induce MSCs and observed osteogenic differentiation (Esposito et al., 2012). Therefore, magnetic flux density is generally limited to 0.1–3 mT, but the optimal density remains unknown. On the other hand, for frequency, MSCs were exposed to PEMFs with different frequencies (5, 25, 50, 75, 100, and 150 Hz), and all groups showed osteogenic differentiation, but with the increase of frequencies, osteogenic differentiation initially enhanced and then peaked at 50 Hz, which decreased until 150 Hz (Luo et al., 2012). Lim et al. also found that PEMF with 50 Hz shows improved osteogenic differentiation compared with those with 10 and 100 Hz (Lim et al., 2013). Therefore, the frequency of PEMF should be limited to 10–150 Hz, and 50 Hz is preferable.

## 3 Osteoinductive Mechanisms of Biophysical Stimuli for Bone Tissue Engineering

When distinctive biophysical stimuli are subjected to MSCs, they will be ultimately loaded by or transferred to either mechanical or electromagnetic stimuli. Thus, mechanotransduction and electrocoupling have been separately proposed to interpret their molecular mechanisms. Considering that both signaling show high comparability and that they simultaneously occur in physiological microenvironment, we propose a new concept of biophysical transduction that integrates mechanotransduction and electrocoupling. Biophysical transduction mainly includes three stages: sensing, transmission, and regulation. According to the sensing pattern of MSCs, biophysical transduction can be further categorized into self-biophysical transduction, cell–matrix biophysical transduction, and cell–cell biophysical transduction.

### 3.1 Self-Biophysical Transduction

**FIGURE 3:**
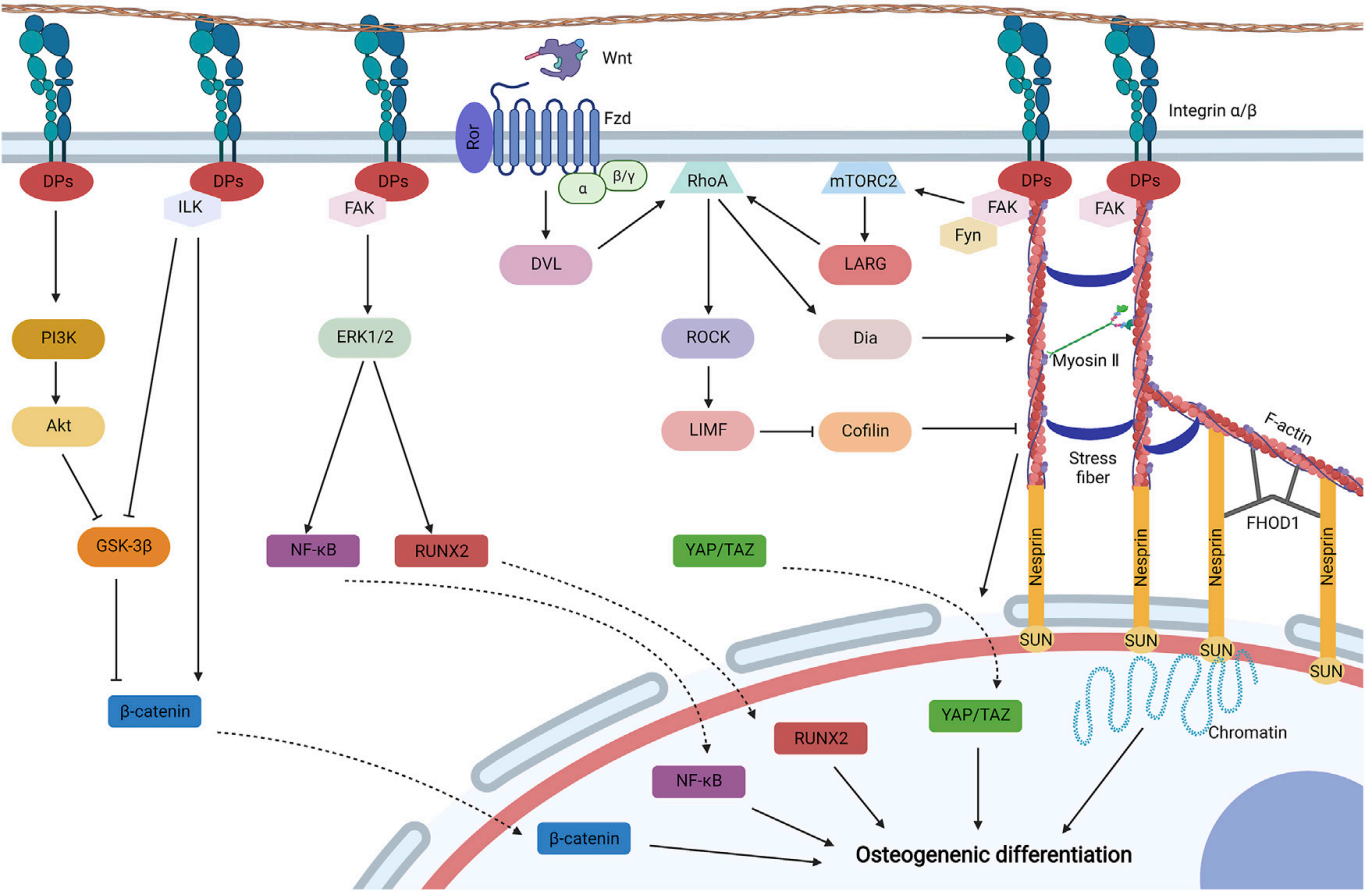
Self-biophysical transduction for mesenchymal stem cell (MSCs) osteogenesis, including biophysical sensitive ion channels, primary cilium, and biophysical sensitive. When MSCs are exposed to biophysical stimuli, various biophysical sensitive ion channels on the plasma membrane may be activated for the influx of Ca^2+^, which include voltage-gated calcium channels (VGCC), stretch-activated calcium channels (SACCs), Piezo, transient receptor potential vallanoid 1 (TPRV1), and TPRV4. TPRV4 is located at high-strain regions, especially primary cilium. Biophysical stimuli can also stimulate the release of Ca^2+^ from the endoplasmic reticulum to the cytoplasm by uncanonical Wnt-Ca2+ signaling. In specific, Wnt ligand binds to the Frizzled (Fzd)/receptor tyrosine kinase-like orphan receptor (Ror) complex to activate Dishevelled (Dvl), which then activates phospholipase C (PLC) to produce inositol 1,4,5-trisphosphate (IP3). IP3 could activate IP3 receptor for the release of Ca^2+^ from the endoplasmic reticulum. The increased Ca^2+^ in the cytoplasm may promote osteogenic differentiation by activating calcineurin/Nuclear Factor of Activated Cells (NF-AT) signaling and initiating extracellular signal-related kinase 1/2 (ERK1/2) by focal adhesion kinase (FAK) to regulate the activity of β-catenin. Biophysical stimuli could regulate the primary cilium containing intraflagellar transport protein 88 (IFT88) for MSC osteogenesis by regulating length or Gpr161/adenylyl cyclase 6 (AC6) signaling, and the activation of AC6 promotes the synthesis of cyclic adenosine 3′,5′-monophosphate (cAMP), which then promotes the osteogenic differentiation of MSCs by Hedgehog (Hb) signaling and proteinkinase A (PKA) signaling. Biophysical stimuli could also promote some long noncoding RNAs (lncRNAs) to inhibit some microRNAs (miRNAs) for osteogenic differentiation. Created with BioRender.com.

Self-biophysical transduction refers to biophysical sensing coupled with transmission and regulation by structures that do not adhere with biomaterials and adjacent cells, which mainly include biophysical-sensitive ion channels and primary cilium. And some biophysical-sensitive ribose nucleic acids (RNAs) are also upregulated by biophysical stimuli to regulate the osteogenic differentiation of MSCs (Figure 3).

#### 3.1.1 Biophysical-Sensitive Ion Channels

A plethora of biophysical-sensitive ion channels exist on the surface of plasma membrane or endoplasmic reticulum, which can be activated by distinctive biophysical stimuli for Ca^2+^ influx to induce the osteogenic differentiation of MSCs. When a sufficiently giant electric field is applied to plasma membrane and generate a large transmembrane potential difference (over 100 mV), voltage-gated calcium channels can be directly activated because of plasma depolarization (Thrivikraman et al., 2018). Electric field and mechanical tension could directly promote the influx of Ca^2+^ by activating stretch-activated calcium channels (Cho et al., 1999; Kearney et al., 2010). In addition, biophysical-sensitive Piezo1 for Ca^2+^ influx could be initiated by mechanical stimuli, such as HP (Sugimoto et al., 2017). Transient receptor potential vallanoid 1 (TRPV1) can be activated by nanovibration (Tsimbouri et al., 2017), whereas TRPV4 can be activated by load-induced FFSS, which is mainly located at high-strain regions (especially primary cilium) (Corrigan et al., 2018; Eischen-Loges et al., 2018). The initiation of these biophysical-sensitive ion channels increases Ca^2+^ concentration in the cytoplasm, which may initiate calmodulin/calcineurin signaling (Kapat et al., 2020). Calcineurin frees the phosphate group from phosphorylated nuclear factor of activated cells (NF-AT), and dephosphorylated NF-AT then shuttles to the nucleus and interacts with other transcription factors to induce the osteogenic differentiation of MSCs (Khare et al., 2020). In addition, increased Ca^2+^ in the cytoplasm initiates protein kinase C and extracellular signal-related kinase 1/2 (ERK1/2), which then regulate the activity of β-catenin to promote MSC osteogenesis (Tsimbouri et al., 2017).

The increase in Ca^2+^ concentration in the cytoplasm can also be mediated by Ca^2+^ release from the endoplasmic reticulum, which may be related to noncanonical Wnt-Ca^2+^ signaling (Bertrand et al., 2020). Biophysical stimuli promote the expression of Wnt (Chen et al., 2019; Fu et al., 2020). Thus, secreted Wnt may interact with a transmembrane receptor Frizzled (Fzd) coupled with receptor tyrosine kinase-like orphan receptor (Ror) to promote the activity of phospholipase C (PLC) by activated Dishevelled (Dvl), and then PLC degrades phosphatidylinositol 4,5-bisphosphate in the cell membrane to obtain inositol 1,4,5-trisphosphate (IP3), which moves to the endoplasmic reticulum and binds to IP3 receptors to promote Ca^2+^ release (Thrivikraman et al., 2018). Moreover, Ca^2+^ channels on the endoplasmic reticulum may be directly modulated by electromagnetic stimuli because of the change in configuration, which also promotes the increase in Ca^2+^ concentration in the cytoplasm (Khare et al., 2020). Therefore, endoplasmic reticulum-derived Ca^2+^ may participate in the osteoinduction of biophysical stimuli.

#### 3.1.2 Primary Cilium

Primary cilium is a biophysical-sensitive organelle based on immotile microtubule and appears from the cytomembrane surface (Delaine-Smith and Reilly, 2012). Primary cilium could sense mechanical and electromagnetic stimuli to induce the osteogenic differentiation of MSCs (Hoey et al., 2012; Chen et al., 2016). The osteoinductive mechanism of primary cilium may be related to its length regulation and ciliary receptors or molecules. One research revealed that topography influences the length of primary, and reduced length promotes the nuclear translocation of β-catenin (McMurray et al., 2013). Thus, specific biophysical stimuli may lower the length of primary cilium for MSC osteogenesis. Gpr161 is a biophysical-sensitive G protein-coupled receptor (GCPR) localized to primary cilium containing intraflagellar transport protein 88 (IFT88), which is essential for cilium formation (Johnson et al., 2021). After being subjected to biophysical stimuli, the GCPR may activate ciliary localized adenylyl cyclase 6 (AC6) for the synthesis of cyclic adenosine 3′,5′-monophosphate (cAMP), which then activates Hedgehog signaling for MSC osteogenesis (Johnson et al., 2018; Johnson et al., 2021). cAMP may also initiate protein kinase A to induce the osteogenic differentiation of MSCs (Johnson et al., 2018). Further studies should focus on other receptors or molecules localized to primary cilium from MSCs and their biophysical transduction and osteoinductive mechanisms.

#### 3.1.3 Biophysical-Sensitive RNAs

After being subjected to biophysical stimuli, MSCs could express some RNAs, including microRNAs (miRNAs) and long noncoding RNAs (lncRNAs), to regulate osteogenic differentiation. One research revealed that tension promotes the expression of lncRNA-MEG3, which then inhibits the expression of miRNA-140-5p for MSC osteogenesis (Zhu et al., 2021). Another research revealed that lncRNA H19 is upregulated to impede miRNA-138, and decreased miRNA-138 may recover the expression of focal adhesion kinase (FAK), which participates in cell–matrix biophysical transduction (Wu et al., 2018). miRNA-132-3p and miR-129-5p are also inhibited when MSCs are subjected to biophysical stimuli for osteogenesis (Hu et al., 2020; Wu et al., 2021). Although biophysical-sensitive RNAs exert critical effects in biophysical regulation, related studies are limited, which need further attention.

### 3.2 Cell–Matrix Biophysical Transduction

**FIGURE 4:**
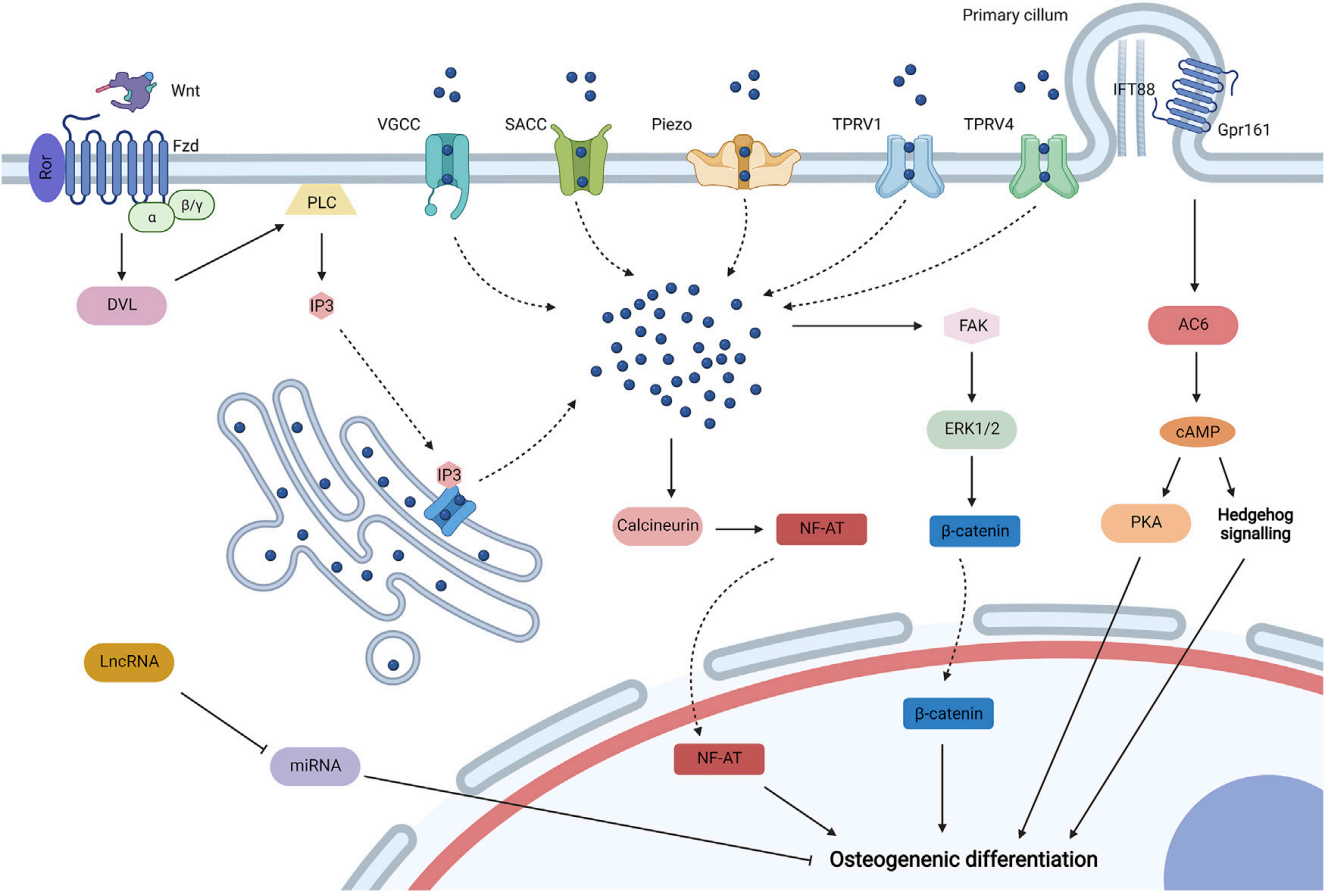
Cell–matrix biophysical transduction for MSC osteogenesis, including integrin signaling and focal adhesion. Integrins generally induce cell adhesion, and integrin signaling may be activated for the osteogenic differentiation of MSCs under biophysical stimuli. Integrin activates phosphatidylinositol 3-kinase (PI3K) to initiate Akt, which then avoids the degradation of β-catenin by inhibiting glycogen synthase kinase-3β (GSK-3β). GSK-3β can be also impeded by integrin linked kinase (ILK) initiated by integrin. Activated ILK can also promote the cytoplastic accumulation of β-catenin by dissociating β-catenin from cadherin. β-catenin is then translocated to the nucleus for osteogenic differentiation. Integrin can also initiate FAK to activate mitogen-activated protein kinase (MAPK) (such as ERK1/2) to phosphorylate transcription factors, such as Runt-related transcription factor 2 (Runx2) and nuclear factor κB (NF-κB), which then shuttles to the nucleus for MSC osteogenesis. Biophysical stimuli can be directly transduced by the structure of focal adhesion-cytoskeleton-nucleus to promote chromatin for gene expression, which is composed of integrin, docking proteins (DPs), recruited FAK, F-actin, myosin II, stress fiber, the Linker of Nucleoskeleton and Cytoskeleton (LINC) complex (nesprin and SUN protein), and formin homology 1/formin homology 2 domain containing protein 1 (FHOD1). The structure is mainly regulated by ras homolog family member A (RhoA) signaling. RhoA can be activated by leukemia-associated Rho guanine nucleotide exchange factor (LARG), which is initiated by the recruitment of Fyn and FAK to initiate mammalian target of rapamycin complex 2 (mTORC2). RhoA can be also activated by initiated Dvl by the binding of Wnt to the Ror/Fzd complex. Activated RhoA initiates LIM kinase (LIMK) by stimulating Rho-associated protein kinase (ROCK), which could inhibit cofilin to avoid F-actin severing. Activated RhoA also promotes the formation and contraction of the structure by initiating Diaphanous (Dia). Furthermore, contracted cytoskeleton may diminish the mechanical resistance for the translocation of Yes-associated protein (YAP) and transcriptional co-activator with PDZ-binding motif (TAZ) to the nucleus for osteogenesis. Created with BioRender.com.

Cell–matrix biophysical transduction refers to biophysical sensing coupled with transmission and regulation by structures that adhere to biomaterials. Cell adhesion is mainly induced and regulated by integrin and focal adhesion (Figure 4).

#### 3.2.1 Integrin Signaling

Integrin is a transmembrane heterodimer that serves as a receptor to bind with specific ligands from external microenvironment (Thompson et al., 2012). When MSCs adhere to biomaterials and subjected to biophysical stimuli, multiple integrin signaling can be activated for the osteogenic differentiation of MSCs. FAK, under biophysical stimuli, may be recruited to integrin and undergo autophosphorylation (Bertrand et al., 2020), which then activates mitogen-activated protein kinases (MAPKs), such as ERK1/2 and P38 (Liu et al., 2014; Niu et al., 2017; Chang et al., 2019). Activated or phosphorylated MAPKs then induce the phosphorylation of transcription factors, such as RUNX2, to promote the osteodifferentiation of MSCs (Chang et al., 2019). Activated ERK1/2 also phosphorylates nuclear factor κB (NFκB), which upregulates integrin β1 in a feedback model and promotes the expression of BMP-2 for osteogenesis (Liu et al., 2011; Liu et al., 2014). Another research revealed that activated ERK1/2 inhibits adipogenesis by downregulating the expression of BMP-4 (Lee et al., 2012). Moreover, biophysical stimuli may activate integrin-linked kinase (ILK) by integrin, which inhibits N-cadherin to release β-catenin and glycogen synthase kinase-3β (GSK-3β) to avoid the degradation of β-catenin. Then, β-catenin shuttles to the nucleus to induce the osteogenic differentiation of MSCs (Niu et al., 2017). Furthermore, integrin could be stimulated by biophysical stimuli to activate Akt by phosphatidylinositol 3-kinase (PI3K), which then activates GSK-3β to inhibit the degradation of β-catenin for MSC osteogenesis (Song et al., 2017; Sun et al., 2018). Osteoinductive mechanisms of integrin signaling by biophysical stimuli can be mainly interpreted as FAK/MAPK signaling, ILK/β-catenin signaling, and PI3K/Akt/β-catenin signaling. In addition, crosslinking exists among distinctive integrin signaling.

#### 3.2.2 Focal Adhesion-Cytoskeleton-Nucleus

In addition to indirect integrin signaling to induce osteogenesis, integrins also participate in the formation of focal adhesion to transfer biophysical stimuli directly to contractible cytoskeleton and nucleus, which is generally known as bundles of clustered integrins (Dalby et al., 2014). At the site of focal adhesion, the cytoplasmic tail of integrin is linked to the (F)-actin cytoskeleton by talin and vinculin, which is stabilized by other docking proteins, including zyxin, actinin, and p130Cas (Bertrand et al., 2020). Adjacent actins generate prestress by the bundling of stress fibers (such as α-actinin) and myosin II (Wang et al., 2009). The tail of actin is linked to the nuclear envelope by the Linker of Nucleoskeleton and Cytoskeleton (LINC) complex, which is composed of nesprin (nesprin1 or nesprin2) and SUN (Bouzid et al., 2019). Moreover, formin homology 1/formin homology 2 domain containing protein 1 binds to multiple sites among nesprin and actin to enhance the association (Birks and Uzer, 2021). Furthermore, SUN proteins bind to lamins to form the lamina, and lamina coupled with G-actin and myosin may assemble into the nucleoskeleton, which connects to chromatin and deoxyribonucleic acid (DNA) (Wang et al., 2009). Therefore, the structure of focal adhesion-cytoskeleton-nucleus allows biophysical stimuli to be transferred from outside to DNA and directly activates gene expression (Wang et al., 2009). When MSCs are subjected to biophysical stimuli, autophosphorylated FAK is recruited to focal adhesion and connects to integrin β by talin and paxillin and promotes cytoskeletal contraction, which then promote MSC osteogenesis (Hao et al., 2015; Bertrand et al., 2020).

Cytoskeletal stabilization and contraction are mainly regulated by biophysical-sensitive ras homolog family member A (RhoA) signaling. And biophysical stimuli could promote the osteogenic differentiation of MSCs by RhoA signaling (Arnsdorf et al., 2009a; Zhao et al., 2015). The activation of RhoA is related to focal adhesion and uncanonical Wnt/RhoA signaling. After biophysical stimuli, kinase Fyn and FAK are recruited to focal adhesion, which synthetically initiate mammalian target of rapamycin complex 2 to activate RhoA, which may be related to the activation of leukemia-associated Rho guanine nucleotide exchange factor (Thompson et al., 2013; Thompson et al., 2018). Moreover, biophysical stimuli could stimulate MSCs to upregulate Wnt5a and Ror2 (Arnsdorf et al., 2009b; Shi et al., 2012). Wnt ligand binds to the complex of Ror and Fzd to initiate Dvl, which could also activate RhoA (Bertrand et al., 2020). The activated RhoA then initiates Rho-associated protein kinase (ROCK) and subsequently LIM kinase (LIMK), which inactivate or phosphorylate cofilin to diminish its effects of severing F-actin (Hayakawa et al., 2011; Bertrand et al., 2020). The structure of focal adhesion-cytoskeleton-nucleus can be also enhanced by the initiation of Diaphanous via RhoA (Bertrand et al., 2020).

Yes-associated protein (YAP) and transcriptional co-activator with PDZ-binding motif (TAZ) are biophysical-sensitive transcriptional activators for MSC osteogenesis. When MSCs are subjected to biophysical stimuli, YAP/TAZ are activated and then translocate to the nucleus, where they interact with various transcription factors to promote osteogenesis (Kim et al., 2014; Qian et al., 2017; Li et al., 2020). The regulation of YAP/TAZ stimulated by biophysical stimuli may undergo a Hippo/LATS-independent signaling pathway (Dupont et al., 2011). Recent studies have revealed that the nuclear translocation of YAP/TAZ is related to the structure of focal adhesion-cytoskeleton-nucleus (Driscoll et al., 2015). When biophysical stimuli are loaded to MSCs, forces cause focal adhesion to the nucleus by the cytoskeleton to open the size of nuclear pores relatively. Thus, the mechanical resistance to transfer molecules is lowered, allowing for YAP/TAZ translocate to the nucleus (Elosegui-Artola et al., 2017).

### 3.3 Cell–Cell Biophysical Transduction

**FIGURE 5:**
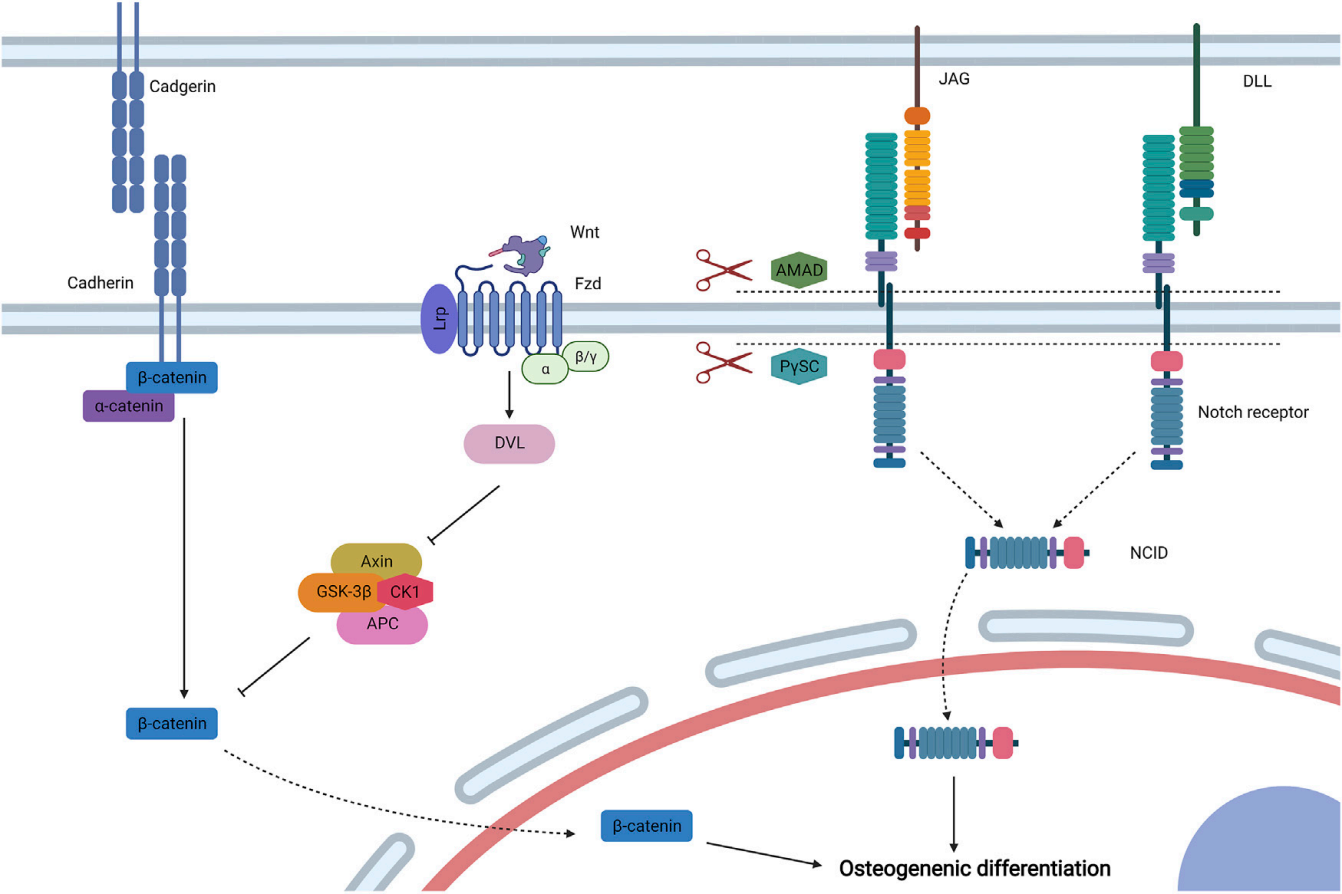
Cell–cell biophysical transduction for MSC osteogenesis: direct interaction. Cadherin is a main molecule to induce direct intercellular interaction, and α-catenin and β-catenin are linked to the cytoplasmic section of cadherin. Under biophysical stimuli, β-catenin dissociates from cadherin to cytoplasm. In addition, β-catenin is mainly regulated by biophysical sensitive canonical Wnt signaling. Wnt binds to the lipoprotein receptor-related protein (Lrp)/Fzd complex and activates Dvl to inhibit the axin/adenomatous polyposis coli/GSK-3β/CK1 (Axin/APC/GSK-3β/CK1) complex to avoid the degradation of β-catenin. Then, β-catenin moves to the nucleus to promote MSC osteogenesis. The osteoinduction of direct cell–cell biophysical transduction can be induced by Notch signaling, which is mediated by Notch receptors and Delta-like (Dll) or Jagged (JAG) ligands. Under biophysical stimuli, two proteases, including ADAM-family metalloproteases and presenilin-γ-secretase complex (PγSC), release Notch intracellular domain (NICD) form Notch receptor, which then moves to the nucleus for osteogenic differentiation. Created with BioRender.com.

**FIGURE 6:**
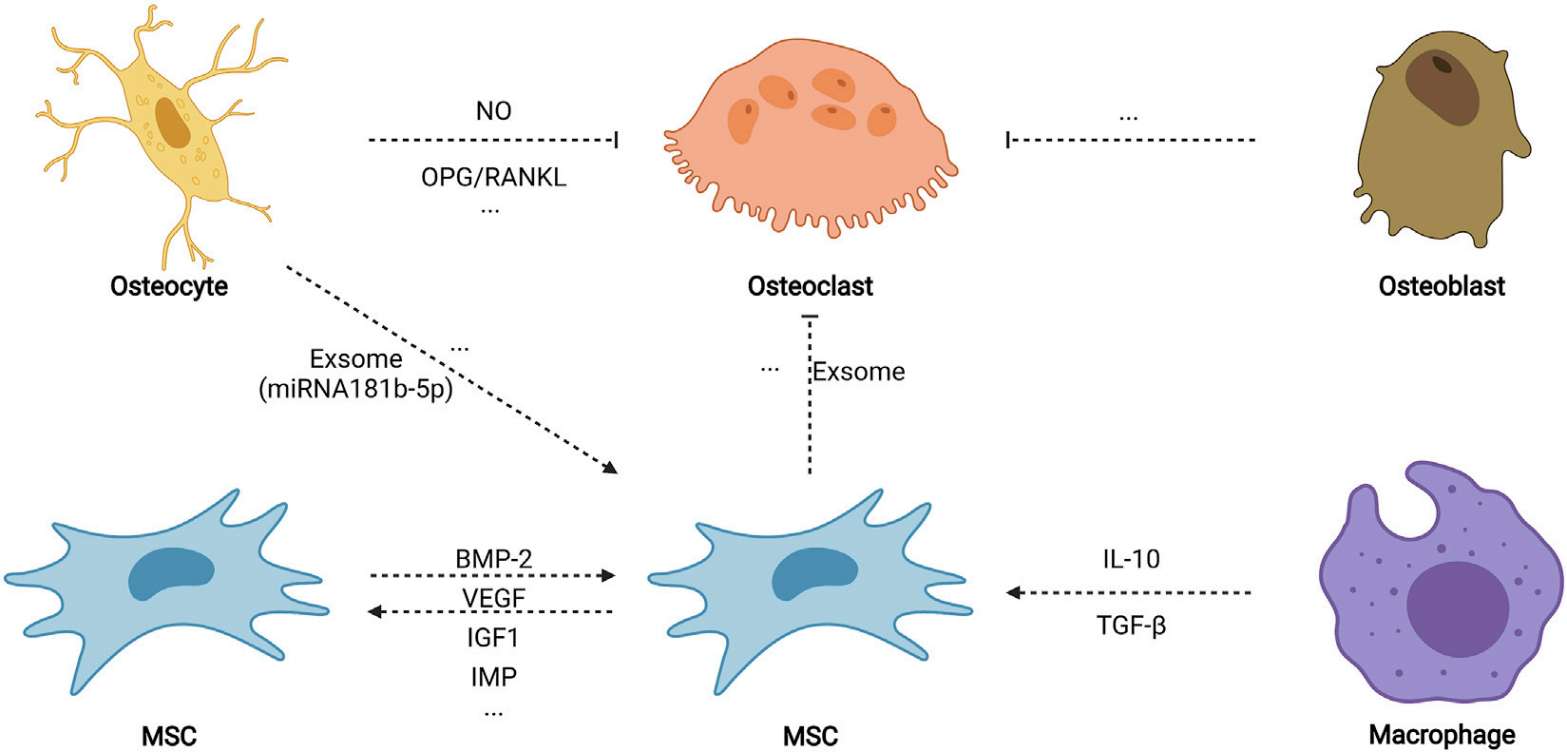
Cell–cell biophysical transduction for MSC osteogenesis: indirect autocrine and paracrine. Indirect cell–cell biophysical transduction is mainly induced by the autocrine and paracrine network. Under biophysical stimuli, MSCs may sensitively secret some autocrine proteins to promote osteogenesis, including BMP-2, VEGF, IGF1, TGF-β, and MIF. MSCs also secrete paracrine factors (such as exosome) to inhibit osteoclastogenesis. Osteocytes release nitric oxide (NO) and relatively more osteoprotegerin (OPG) than NF-κB ligand (RANKL) to impede osteoclastogenesis. Paracrine factors from osteoblasts also diminish osteoclastogenesis. Moreover, paracrine factors (such as exosome containing miRNA181b-5p) stimulate the osteogenic differentiation of MSCs. Biophysical stimuli also stimulate macrophage (phenotype 2) to promote MSC osteogenesis. Created with BioRender.com.

Cell–cell biophysical transduction refers to biophysical sensing coupled with transmission and regulation by direct contact by structures from adhering with adjacent cells (cadherin and Notch receptor) (Figure 5). Bioactive factors under biophysical stimuli by the autocrine and paracrine network could also exert effects via indirect interaction (Figure 6).

#### 3.3.1 Adhesion Junction

Intercellular adhesion junction is directed by calcium-dependent cadherins, and MSCs express neural (N-) cadherin and epithelial (E-) cadherin (Qin L. et al., 2020). The classical structure of cadherins can be divided into three domains: an extracellular domain to direct intercellular adhesion, a single-pass transmembrane domain, and a cytoplasmic domain to bind multiple proteins including β-catenin and α-catenin (Leckband and de Rooij, 2014). When MSCs are subjected to mechanical stimuli (such as load-induced FFSS) or electromagnetic stimuli (such as PEMF), β-catenin disassociates from N-cadherin or E-cadherin to the cytoplasm, respectively, which then moves to the nucleus to induce osteogenic differentiation (Arnsdorf et al., 2009b; Zhang et al., 2020).

The β-catenin signaling pathway is generally regulated by canonical Wnt signaling, which shows biophysical sensitivity. When MSCs are subjected to electromagnetic stimuli for osteogenesis, the expression levels of Wnt, low-density lipoprotein receptor-related protein (Lrp), and β-catenin are upregulated, suggesting that canonical Wnt signaling exerts critical effects in osteogenic differentiation (Chen et al., 2019; Fu et al., 2020). Secreted Wnt binds to the complex formed by Fzd and LRP 5/6, which then activates Dvl to impede the phosphorylation and degradation of β-catenin mediated by the axin/adenomatous polyposis coli/GSK-3β/CK1 (Axin/APC/GSK-3β/CK1) complex (Schupbach et al., 2020). Then, β-catenin shuttles to the nucleus to activate osteogenic Runx2 and osterix (Benayahu et al., 2019). The activation of canonical Wnt signaling by biophysical stimuli could also inhibit MSC adipogenesis by downregulating the expression of peroxisome proliferator-activated receptor γ (Sen et al., 2008; Case et al., 2013).

#### 3.3.2 Notch Signaling

Exerting critical effects on stem cell specification and bone development, Notch signaling is an intercellular conversed pathway that is activated by a surface ligand [Delta-like (Dll)1, 3, or 4 and Jagged (JAG)1 or 2] from adjacent cells to bind their Notch receptor (Notch1, 2, 3, or 4) (Bagheri et al., 2018). After initiation, two types of proteases (ADAM-family metalloproteases and presenilin-γ-secretase complex) exert effects at an activated Notch receptor to release the Notch intracellular domain (NICD). The NICD then forms a complex with the DNA-binding CSL protein after translocating to the nucleus, which recruits coactivator Mastermind to transcript related genes. When MSCs are subjected to PEMF, Notch-4 receptor, Dll-4 ligand, and related genes (Hey1, Hes1, and Hes5) are upregulated, and inhibitors of Notch signaling diminish the osteoinducutive effects of PEMG (Bagheri et al., 2018). Thus, Notch signaling is involved in the osteogenic differentiation of MSCs stimulated by electromagnetic signaling. Another research also reported that mechanical stimuli could initiate JAG1-mediated Notch signaling to promote MSC osteogenesis, which is controlled by the inhibition of endogenous histone deacetylase 1 (Wang et al., 2016a).

#### 3.3.3 Autocrine and Paracrine Network

After being subjected to biophysical stimuli, MSCs secrete various biophysical-sensitive molecules, including BMP-2 (Liu et al., 2011; Rui et al., 2011), vascular endothelial growth factor (Lee et al., 2017), insulin-like growth factor 1 (Tahimic et al., 2016), transforming growth factor-β (TGF-β) (Li et al., 2015b), and migration inhibitory factor (Yuan et al., 2016), which could biochemically promote MSC osteogenesis by autocrine. In addition, bone regeneration *in vivo* is an extremely complex process that involves osteogenesis, angiogenesis, osteoclastogenesis, neurogenesis, and immune regulation; thus, a paracrine network possibly stimulates osteogenesis among various cells when subjected to biophysical stimuli. Paracrine factors from stimulated osteocytes promote the osteogenic differentiation of MSCs but do not induce the osteogenic differentiation of osteoblasts (Hoey et al., 2011; Brady et al., 2015). Another study found that exosomes containing miRNA181b-5p from stimulated osteocytes enhance the osteogenic differentiation of PDLCs by BMP-2/RUNX2 (Lv et al., 2020). Stimulated osteocytes also secrete nitric oxide and relatively more osteoprotegerin than NF-κB ligand (RANKL) to inhibit osteoclastogenesis (Tan et al., 2007; You et al., 2008). Moreover, exosomes from stimulated MSCs impede osteoclastogenesis by weakening the activity of NF-κB signaling (Xiao et al., 2021). Although paracrine factors from stimulated osteoblasts fail to promote the osteogenic differentiation of MSCs, they inhibit the formation of osteoclasts (Tan et al., 2007). Furthermore, Dong et al. (2021) found that tension could activate macrophages to M2 phenotype, secrete anti-inflammatory factors (such as TGF-β and interleukins-10), and promote the nuclear translocation of YAP to generate BMP-2 for the osteogenic differentiation of MSCs. Altogether, the autocrine and paracrine network may ultimately promote the osteogenic differentiation of MSCs *in vivo*, which further promote bone repair.

## 4 Applications of Biophysical Stimuli for Bone Tissue Engineering

For critical bone defects, bone tissue engineering is committed to replace or surpass autografts for surgical bone repair. Given their osteoinductive properties, distinctive biophysical stimuli can be used as the fourth pillar to improve traditional bone tissue engineering for bone healing. Biophysical stimuli can be integrated to bone tissue engineering by three methodologies: preconstructed scaffolds with osteoinductive properties, TEBGs, and postoperative biophysical stimuli loading strategies.

### 4.1 Preconstructed Scaffolds With Osteoinductive Properties

Biomaterial scaffolds comprise an important pillar for bone tissue engineering, but most current scaffolds are used to provide osteoconductivity without osteoinductivity. Thus, most studies focused on the introduction of bioactive molecules to scaffolds by various strategies. However, these methods fail to construct scaffolds with multi-environment for different regenerative purposes. Thus, preconstructed scaffolds by biophysical stimuli show great promise for novel bone tissue engineering because these scaffolds show inherent osteoinductivity, which also support the construction of multi-microenvironment. Preconstructed scaffolds include stiffness-improved scaffolds, topography-modified scaffolds, piezoelectric scaffolds, and magnetically actuated scaffolds.

#### 4.1.1 Stiffness-Improved ECM-like Scaffolds

ECM-like scaffolds are those scaffolds that are structurally analogous to natural nanoscale natural ECM, which can be obtained by electrospinning or hydrogelation. However, the application of ECM-like scaffolds is generally limited by their unsatisfying stiffness. Thus, various strategies, including chemical and physical crosslinking, have been developed to enhance their rigidity. Chemical crosslinking improves stiffness by introducing chemical crosslinkers (such as genipin) and specific chemical groups, whereas physical crosslinking enhances rigidity by constructing an interpenetrating network or physical crosslinkers, such as inorganic particles and graphene (Zhang et al., 2016). When the stiffness of organic scaffolds is improved, these scaffolds could even stimulate the osteogenic differentiation of MSCs without osteogenic molecules. For example, elastin-like polypeptides (ELPs) are recombinant biomaterials that could assemble into organic scaffolds when the temperature reaches over inverse phase transition temperature (Ding et al., 2020). Glassman et al. (2016) utilized telechelic oxidative coupling to chemically interlink or oxidate ELPs containing cysteine, and the stiffness of ELP scaffolds is obviously enhanced, varying from 5 kPa to over 1 MPa. When seeded on the toughened scaffolds, MSCs could be induced to undergo osteogenic differentiation without bioactive molecules. Raftery et al. introduced chitosan to collagen to construct organic scaffolds with an interpenetrating network and found that the modulus is improved and that the interpenetrating scaffold (collagen/chitosan ratio 75/25) could dramatically promote calcium deposition and sulfated glycosaminoglycan production (Raftery et al., 2016). Thus, stiffness-improved scaffolds may be superior to unoptimized organic scaffolds.

#### 4.1.2 Topography-Modified Scaffolds

Modifying topography is another methodology for osteoinductive preconstructed scaffolds. The outer and inner surfaces can be modified for different regenerative purposes. Outer topography-modified scaffolds are those scaffolds whose outer surface are modified by nanopattern with controlled disorder, which could promote osteointegration and avoid the formation of fibrous tissue at the surface of bone implants (Dobbenga et al., 2016). For example, Silverwood et al. (2016) utilized a block-copolymer templated anodization technique to modify the outer surface of titania scaffolds with nanopillars (height 15 nm) and then transplanted modified titania and polished titania to rabbit femurs. Compared with the flat titania, the outer topography-modified titania with 15 nm nanopillars show higher bone to implant contact (20%) (Silverwood et al., 2016). Meanwhile, inter topography-modified scaffolds are those scaffolds whose porous inner surface are modified by controlled nanotopography. For example, Wang et al. used cold atmospheric plasma to treat or modify the inter topography of 3D printed poly-lactic-acid scaffolds, which could improve nanoscale surface roughness from 1.20 to 10.50 nm (1 min treatment), 22.90 nm (3 min treatment), and 27.60 nm (5 min treatment) (Wang et al., 2016b). And modified scaffolds could improve cell adhesion and proliferation when compared with unmodified scaffolds, showing great potential for bone tissue engineering (Wang M. et al., 2016). Further studies should focus on the osteogenic effects of the modified inter surface, and related treating techniques should also be developed.

#### 4.1.3 Piezoelectric Scaffolds

Natural bone is an electricity-generated tissue because of piezoelectric collagen (Yu et al., 2017). Thus, piezoelectric scaffolds are developed to provide electric stimuli for bone regeneration, which include piezoelectric polymers such as PEDOT (Iandolo et al., 2016) and poly (vinylidene fluoride), piezoelectric ceramics such as BaTiO_3_ (Polley et al., 2020) and K_0.5_Na_0.5_NbO_3_ (Yu et al., 2017), and scaffolds containing piezoelectric particles such as piezoelectric ceramic particles (Mancuso et al., 2021) and nylon-11 nanoparticles (Ma et al., 2019)). When piezoelectric scaffolds are subjected to external mechanical stimuli (physiologically or additionally), electric stimuli will be induced for bone regeneration (Tang et al., 2017). Osteogenic differentiation can be obtained by high voltage output form piezoelectric scaffolds (Damaraju et al., 2017). However, the piezoelectricity of scaffolds should be optimized for practical applications. For example, Zhang et al. utilized annealing treatment to control β phase contents, thus constructing piezoelectric poly (vinylidene fluoridetrifluoroethylene) [P(VDF-TrFE)] membranes with different surface potentials (−78 and −53 mV) (Zhang et al., 2018). When transplanted to critical calvarial defects of rats, all groups could promote bone regeneration, but P(VDF-TrFE) membrane with −53 mV surface potential is better because it dramatically promotes faster bone regeneration with more mature bone structure than unpolarized group and that with −78 mv surface potential (Zhang et al., 2018). Furthermore, the osteoinductive effects of piezoelectric scaffolds can enhance additional mechanical stimuli. Piezoelectric scaffolds were fabricated by modifying porous Ti6Al4V scaffolds (pTi) with BaTiO_3_, and then piezoelectric pTi/BaTiO_3_ or pure pTi were transplanted to rabbit radius bone defects with a length of 13 mm (Fan et al., 2020). Results showed that pTi/BaTiO_3_ with or without LIPUS promotes more bone regeneration than pure pTi, but LIPUS treatment could further enhance osteointegration and osteogenesis (Fan et al., 2020).

#### 4.1.4 Magnetically Actuated Scaffolds

Magnetically actuated scaffolds are generally constructed by introducing magnetic particles (usually magnetite) to other biomaterial scaffolds (Santos et al., 2015). When magnetically actuated scaffolds are exposed in magnetic field, the deformation of scaffolds stimulates the differentiation of stem cells (Spangenberg et al., 2021). Aldebs et al. (2020) used PEGF (1 mT, 15 Hz) to stimulate ADSCs for 7 days seeded in RADA16 self-assembling peptide hydrogel containing magnetic particles and found that PEGF coupled with magnetic particles could promote the osteogenic differentiation of ADSCs without spoiling cell viability. Moreover, magnetic nanoparticles can be encapsuled to piezoelectric scaffolds, which could provide magnetomechanical and electromagnetic stimuli (Fernandes et al., 2019). Zhang et al. fabricated magnetically actuated scaffolds with magnetic effects by simultaneously incorporating two magnetic particles: positive CoFe_2_O_4_ (CFO) and negative Tb_x_Dy_1-x_Fe_2_ alloy (TD) to piezoelectric P(VDF-TrFE) (Zhang et al., 2020). A CFO/TD ratio of 4:1 could dramatically improve surface potential when scaffolds are exposed to magnetic field. Then, MSCs were seeded on the film for 7 or 14 days, and a magnetic field was applied at the 4^th^ or 8^th^ day. Results showed that magnetic field could dramatically stimulate the osteogenic differentiation of MSCs in the 14-days culture, but osteogenic differentiation was not observed at the 4-days culture, suggesting that magnetic field-coupled magnetic particles could promote osteogenesis, but sufficient exposure time is needed (Zhang et al., 2020). Although magnetically actuated scaffolds show satisfying biocompatibility when transplanted *in vivo*, additional evidence is needed about bone defect repair *in vivo* by magnetically actuated scaffolds coupled with magnetic field.

### 4.2 Tissue Engineered Bone Grafts by *in vitro* Bioreactors

In addition to prefabricated osteoinductive scaffolds by biophysical stimuli, they can be directly exerted by *in vitro* bioreactors to construct TEBGs, artificial autografts with anatomically matched shape and size (Grayson et al., 2010). TEBGs are generally fabricated by a combination of a biomaterial scaffold, autogenous stem cells, osteogenic supplements, and *in vitro* bioreactors providing biophysical stimuli (Fröhlich et al., 2010).

Among various *in vitro* bioreactors, load-induced FFSS-based bioreactors have been widely used to construct TEBGs, especially perfusion bioreactors. The efficiency of TEBGs for bone tissue engineering was initially verified by ectopic bone formation in animal models. Hosseinkhani et al. mixed MSC suspension with peptide amphiphile (PA) solution to form PA nanofiber hydrogel containing MSCs, which was then infiltrated to collagen/poly (glycolic acid) sponge for static or prefusion culture (Hosseinkhani et al., 2006). After 3 weeks of *in vitro* culture, both groups were transplanted to the back subcutis of rats for 8 weeks, and results showed that the TEBGs obtained by perfusion culture promote homogenous and robust bone regeneration when compared with those by static culture (Hosseinkhani et al., 2006). Moreover, TEBGs for critical bone defects have been verified in clinically relevant pig bone defect models. An image-guided personalized strategy was utilized to construct TEBGs by 3 weeks of invitro culture (Bhumiratana et al., 2016). TEBGs are constructed by a combination of decellularized bone matrixes (DBMs), autogenous ADSCs, osteogenic medium, and a perfusion-based bioreactor, and immure bone formation is observed after 3 weeks of perfusion culture. Then, TEBGs or acellular DBMs were transplanted to bone defects of mature Yucatán minipigs for 6 weeks, and results revealed that TEBGs show improved bone formation and vascularization compared with untreated defects and acellular scaffolds (Bhumiratana et al., 2016). Therefore, TEBGs show great promise for critical bone defects, which ideally integrate four pillars of bone tissue engineering.

TEBGs may be the second-generation autogenous bone grafts for surgical bone repair. Thus, various methods have been developed to improve the effectiveness of TEBGs, which are based on four hierarchies. The first level is to optimize perfusion profile, which includes perfusion duration and perfusion model. Dynamic culture for at least 2 weeks is needed to fabricate TEBGs (Mitra et al., 2017). In addition, sequential application of continuous perfusion and intermittent perfusion may be better than single model (Correia et al., 2013). The second level is to optimize bioreactor system medium. For example, marine hemoglobin could be incorporated to perfusion medium to deliver sufficient oxygen, which enhances the proliferation and osteogenic differentiation of MSCs in perfusion bioreactors (Le Pape et al., 2018). The third level is to optimize seed cells. For example, BMSCs may be superior to ADSCs because of better potential for osteogenesis (Wu et al., 2015). In addition, co-culture of multiple cells may be superior to single cell culture for the fabrication of TEBGs (Sawyer et al., 2020), but multiple cells must be obtained from autogenous stem cells. Thus, co-culture of osteogenically indued stem cells and angiogenic-induced stem cells shows great promise. Furthermore, the fourth level is to optimize biomaterial scaffolds. Hydrogels can be used to modify other biomaterials because hydrogels could improve cell retention (Yu et al., 2012). In addition, bioactive molecules can be introduced to biomaterial scaffolds to provide sustained-release osteoinductive signaling (Panek et al., 2019).

In addition to perfusion bioreactors, load induced-FFSS-based bioreactors include rotating wall vessel bioreactors, spinner flask bioreactors, and biaxial rotating bioreactors, among which biaxial rotating bioreactors may surpass other uni-axial perfusion bioreactors because they generate homogenous FFSS and promote robust osteogenesis (Singh et al., 2005; Zhang et al., 2010a). TEBGs fabricated by biaxial rotating bioreactors induce ectopic bone regeneration (Zhang et al., 2009) and promote the healing of critical femoral defects of rats (Zhang et al., 2010b).

Other bioreactors include compression bioreactors (Ravichandran et al., 2017), tension bioreactors (Carroll et al., 2017), nanovibration bioreactors (Wu et al., 2020), ultrasound bioreactors (Wang M. et al., 2016), and electromagnetic bioreactors (Fernandes et al., 2019), which show promise to construct TEBGS, but they are limited to transport nitrogen, oxygen, and growth factors. Therefore, other bioreactors can be synthetically used with load induce-FFSS based bioreactors to design multi-biophysical stimuli bioreactors. For example, a multimodal bioreactor was designed which could provide compression and load-induced FFSS, and osteogenic differentiation is improved when compared with static culture and culture with single biophysical stimuli when the system is used to dynamically culture MSCs (Ravichandran et al., 2018). Further evidence is needed about TEBGs fabricated by multimodal bioreactors to repair critical bone defects.

### 4.3 Postoperative Biophysical Stimuli Loading Strategies

Postoperative biophysical stimuli loading strategies are those methods that use noninvasive methods to generate biophysical stimuli to stimulate bone regeneration after surgery, including distraction osteogenesis (DO) (Shah et al., 2021), LMHFV (Steppe et al., 2020), LIPUS (Hannemann et al., 2014), and PEMF (Wang et al., 2019). These strategies have been clinically used to promote the healing of fractures; thus, their use can be extended to the treatment of critical bone defects. For example, Parmaksiz et al. transplanted DBM and DBM containing magnetic particles to bilateral critical-size cranial defects of rats with or without PEMF exposure, and results revealed that exposed groups of DBM and DBM containing magnetic particles show improved bone regeneration and reduced fibrous formation compared with unexposed groups (Parmaksiz et al., 2021). Yan et al. also verified that postoperative LIPUS could promote the healing of rabbit femurs defects (Yan et al., 2016). Therefore, postoperative biophysical stimuli loading strategies show great promise in bone tissue engineering, but they remain poorly explored. Bone defects are usually limited to local regions. Thus, small loading devices are also highly developed for clinical bone repair. When postoperative biophysical stimuli loading strategies are used in combination of other pillars of bone tissue engineering, their clinical complications or drawbacks may diminish. For example, DO is a long-term treatment that may cause infections or delay, but it shows great promise for bone regeneration when combined with osteoinducive Mg nail, which could reduce the treatment time of traditional DO (Ye et al., 2021).

## 5 Discussion

Considering the inefficiency of current biomaterials and bioactive molecules, biophysical stimuli are critical to be applied as the fourth pillar of bone tissue engineering. In addition, biophysical stimuli should not be only limited to external mechanical stimuli, but in a more comprehensive concept, the fourth pillar can also include internal structural stimuli, acoustic stimuli, and electromagnetic stimuli. Although distinctive biophysical stimuli are based on different physical properties, they will be transferred to mechanical or electromagnetic stimuli. Given that both show reminiscent signal pathways for MSC osteogenesis, a novel concept of biophysical transduction is proposed to incorporate mechanotransduction and electrocoupling to interpret the osteoinductive mechanisms of biophysical stimuli for osteogenic differentiation. Biophysical transduction can be divided into three stages: sensing, transmission, and regulation. Depending on sensing pattern, biophysical transduction can be categorized into self-biophysical transduction, cell–matrix biophysical transduction, and cell-cell biophysical transduction. Furthermore, biophysical stimuli, as the fourth pillar of bone tissue engineering, can be used by fabricating preconstructed scaffolds with osteoinductive properties and TEBGs or by employing postoperative biophysical stimuli loading strategies.

While biophysical stimuli show promising potential to be used as the fourth pillar of bone tissue engineering, ideal biophysical stimuli are needed to promote cell recruitment, proliferation, osteogenesis, angiogenesis, neurogenesis, and immune regulation. Osteoinductive windows of biophysical stimuli need to be comprehensively considered and optimized. The synergy effects of multiple biophysical stimuli should be also explored for bone regeneration. Moreover, specific osteoinductive mechanisms of biophysical stimuli need further investigation. The interaction between mechanotransduction and electrocoupling should be probed to interpret their similarity. Whether or not other cell adhesion receptors and ligands participate in cell–matrix biophysical transduction remains unknown. The autocrine and paracrine network of cell–cell biophysical transduction also needs to be refined. *In vivo* preclinical evidence about preconstructed scaffolds may be obtained, especially for piezoelectric scaffolds and magnetically actuated scaffolds. Novel *in vitro* bioreactors may be designed to diminish complexity and volume. Further studies may focus on the application of postoperative biophysical stimuli loading strategies for the treatment of critical bone defects. Specific parameters using model and duration may be established.
